# Stay-at-home orders due to the COVID-19 pandemic are associated with elevated depression and anxiety in younger, but not older adults: results from a nationwide community sample of adults from Germany

**DOI:** 10.1017/S0033291720003438

**Published:** 2020-09-08

**Authors:** Christoph Benke, Lara K. Autenrieth, Eva Asselmann, Christiane A. Pané-Farré

**Affiliations:** 1 Department of Psychology, Clinical Psychology and Psychotherapy, Philipps University of Marburg, Gutenbergstraße 18, Marburg 35032, Germany; 2 Department of Psychology, Personality Psychology, Humboldt-Universität zu Berlin, Unter den Linden 6, Berlin 10099, Germany

**Keywords:** Coronavirus, lockdown, public health measures

When the COVID-19 pandemic started to spread around the globe in early 2020, public health measures such as stay-at-home orders were taken worldwide to prevent a further dissemination of the virus. Stay-at-home orders promise to be highly effective in terms of physical distancing. However, especially in vulnerable groups, they may also promote loneliness and social isolation and thus increase the risk of depression and anxiety (Aleman & Sommer, 2020; Brooks et al., 2020; Holmes et al., 2020). Identifying who is at increased risk for depression and anxiety during stay-at-home orders is thus particularly important for developing targeted preventions and early interventions.

Between 17th April and 15th May 2020, an online survey was conducted in a nationwide community sample of adults from Germany (*N* = 4335; 75.8% women and 24.2% men; *M* = 40.50 years, s.d. = 12.45 years). The sample was drawn using convenience sampling methods (e.g. via social media and email). All participants provided informed consent. The study was approved by the local Ethics Committee of the University of Marburg. Overall, 48.3% (*N* = 2023) reported to be under an officially announced stay-at-home order. Individuals who did vs. did not report to be under a stay-at-home order were slightly younger (*M* = 38.9 *v. M* = 41.8 years), *t*(4182) = 7.583, *p* < 0.001, had a higher educational level, χ^2^ = 12.791, *p* < 0.001, and more often reported to be single, χ^2^ = 4.437, *p* = 0.035. To test whether the association of a stay-at-home order with depressive and anxiety symptoms varied by sociodemographic and COVID-19-related variables, logistic regressions with interaction terms (adjusted for gender and age) were conducted. The alpha level was set at 0.05.

Consistent with previous findings from the USA (Tull et al., 2020), participants who did vs. did not report to be under a stay-at-home-order were at higher risk to suffer from depression [odds ratio (OR) 1.274, 95% confidence interval (CI) 1.115–1.457, *p* < 0.001] or anxiety (OR 1.256, 95% CI 1.099–1.435, *p* = 0.001). Specifically, 34.5% of those with and 27.8% of those without stay-at-home orders were affected by at least moderate depression (PHQ-9 ⩾ 10). Moreover, 33.9% of those with and 28.0% of those without stay-at-home orders were affected by at least moderate anxiety (DSM-5 Level 2 cross-cutting symptom measure for anxiety ⩾ 20).

Importantly, this risk-association varied by age (depression: OR 0.985, 95% CI 0.974–0.996, *p* = 0.009; anxiety: OR 0.987, 95% CI 0.976–0.998, *p* = 0.019): only in younger individuals (aged 18–34 years; depression: OR 1.531, 95% CI 1.241–1.889, *p* < 0.001; anxiety: OR 1.495, 95% CI 1.205–1.854, *p* < 0.001), but not in middle-aged and older individuals (aged 35–49, 50–64 and 65+; all *p* values > 0.05), stay-at-home orders were associated with depression and anxiety. Specifically, 43.2% of younger adults with stay-at-home orders and 33.2% of younger adults without stay-at-home orders were affected by at least moderate depression. Moreover, 40.0% of younger adults with stay-at-home orders and 30.8% of younger adults without stay-at-home orders were affected by at least moderate anxiety. Other sociodemographic factors such as gender, education, employment status, living with underaged children, and living alone as well as previous mental health problems and belonging to a risk group for a severe course of COVID-19 did not interact with stay-at-home orders in predicting depression and anxiety (all *p* values > 0.05).

Our findings suggest that especially younger adults are a vulnerable group for depression and anxiety due to lockdown measures. These results are in line with recent longitudinal findings from the UK that younger adults were at increased risk for mental health problems during the COVID-19 lockdown (Pierce et al., 2020). The present results suggest that stay-at-home orders might significantly contribute to this decline in mental health in younger adults.

How might these findings be explained? The developmental period of young adulthood is a time frame in which social relationships and social activities with peers play a particularly important role (Reitz, Zimmermann, Hutteman, Specht, & Neyer, 2014): young adults often engage in large-scaled social events such as parties and concerts, which are particularly hit by the crisis. Moreover, many young people are living in single households, have not yet started a stable relationship or family, but are still establishing such ties (Asselmann & Specht, 2020). Therefore, stay-at-home orders might disrupt their social life in a particularly serious way. Consistently, previous research found that younger age groups tend to experience higher levels of loneliness – in general (Luhmann & Hawkley, 2016) as well as during the COVID-19 pandemic (Luchetti et al., 2020). Taken together, these reasons might explain our result that especially younger individuals were at increased risk for depression and anxiety symptoms in response to stay-at-home orders. Future studies might examine these potential underlying mechanisms in higher detail and identify modifiable protective factors, which might reduce the negative impact of stay-at-home orders on mental health in younger adults. Above and beyond previous recommendations (Aleman & Sommer, 2020), the results of this study call for more attention to mental health problems of younger adults who are affected by stay-at-home orders.
